# Group Appeals of Parties in Times of Economic and Identity Conflicts and Realignment

**DOI:** 10.1177/00323217221123147

**Published:** 2022-09-24

**Authors:** Simon Stuckelberger, Anke Tresch

**Affiliations:** 1 Faculty of Social Sciences, Goethe University Frankfurt am Main, Germany; 2 Institute of Political Studies, FORS and University of Lausanne, Lausanne, Switzerland

**Keywords:** group appeals, parties, realignment, cleavages, political communication

## Abstract

Party–group relations are today characterized by various forms of alignments. These include the persistence of traditional class alignments, the realignment of economic groups due to identity politics and alignments of groups at the centre of identity politics. This study analyses the group-based messaging of parties in relation to these three groups. We argue that, contrary to the catch-all party thesis, positive and negative group appeals of parties are (still) structured by parties’ support among social groups and by groups either liked or disliked by their voters. Our argument is tested through a content analysis of election materials in Germany, Switzerland and the Netherlands, combined with survey data. The results indicate that group appeals are indeed shaped by electoral support among social groups and attitudes towards them. Results also suggest that the former effect is present for traditionally aligned economic and identity politics groups, but not for realigned economic groups.

## Introduction

In *Political Man*, Lipset (1960: 220) famously emphasized the centrality of class conflict for party competition, arguing that parties are the ‘translation of class struggle’ and that ‘their appeals and their support suggest that they do represent the interests of different classes’. Party competition was then clearly structured by an economic cleavage between right-wing parties representing the interests of higher economic classes and left-wing parties advocating the interests of working-class constituents. Crucially, as Lipset (1960) stressed, the class base of parties was also expressed in *their appeals*: political parties explicitly underlined their ties with their core constituencies and did not hesitate to criticize classes on the other side.

Yet party competition has since witnessed the dampening of the class conflict and the emergence of a particularly salient cultural conflict. This new cultural divide stems from identity politics that stress the recognition and protection of groups such as women and sexual or ethnic minorities on the left and on the right by an emphasis on a national identity and the threat posed by the EU and immigration (Bornschier, 2015; Kriesi et al., 2008, 2012). This two-dimensional conflict structure has transformed party–group relations, resulting in three types of aligned groups. First, *traditionally aligned economic groups* stand for the endurance of the class conflict, which include employers aligned with their traditional class-allies and who vote mainly by taking economic considerations into account (Oesch and Rennwald, 2018). Second, the *realigned economic groups*, who stand for the transformation of the ties between economic groups and parties in the wake of the emerging cultural conflict. Despite some cross-country variations, the working class and people with lower levels of education have become the stronghold of the radical right, while the left now counts on the support from parts of the middle class and the highly educated (Kriesi et al., 2008, 2012; Oesch and Rennwald, 2018; Stubager, 2013). These realigned groups tend to cast their ballots based on cultural attitudes, with issues such as immigration or minority rights featuring prominently. Finally, the *Identity Politics (IP) groups* concern alignments of newly politicized groups. The politicization of identities of women and immigrants has contributed not only to the described realignment of other groups but also to their alignment with left-wing parties (e.g. Inglehart and Norris, 2000; Strijbis, 2014).

In the literature on party competition, these transformations have mainly been assessed from the standpoint of programmatic appeals, showing for instance that parties have shifted their attention onto cultural issues, at the expense of the economic dimension and redistributive policies (Kriesi et al., 2012). However, party–voter realignments and the rise of identity politics also raise important questions for the group-based messaging parties engage in. Are party appeals, as described by Lipset (1960), still representative of the interests of different classes or social groups? Recent studies that have focused primarily on social democratic parties and economic groups suggest that parties nowadays increasingly refer to the same broad and neutral groups, considering all classes as potential constituencies (Damhuis and Karremans, 2017; Evans and Tilley, 2017; Horn et al., 2021). By contrast, we argue that parties still strongly diverge from one another in terms of their group-based messaging, depending on their electoral support among particular social groups and the attitudes of their voters towards such groups.

First, we contend that in the case of traditionally aligned economic groups and IP groups, the electoral support of parties among these groups is what structures group-based appeals. In other words, parties with a higher vote share among traditionally aligned groups and IP groups should be more likely to target them with positive group appeals. But where realigned economic groups are concerned, we do *not* expect parties to compete for their votes with class-based appeals, given that these groups vote based on issues of cultural conflict rather than their economic identities. For example, the literature suggests that the electoral support of workers for radical right parties is due to the ‘nativist’ agenda pursued by these parties (e.g. Bornschier and Kriesi, 2014). Radical right parties therefore have little reason to appeal to such voters in terms of members of the working class. Put differently, the vote share of parties among realigned economic groups should be unrelated to their appeals towards said groups.

Second, we argue that parties respond to their voters’ positive and negative attitudes towards social groups. If a party’s voters have positive or negative feelings towards specific groups, such as lesbian, gay, bisexual and transgender (LGBT) people or immigrants, the party is expected to tap into these group sentiments with corresponding statements, even if they do not constitute an important voter base.

We therefore contend that parties operate according to two different motivations when using group appeals: they either target social groups among their voters with explicit statements of support, an idea we refer to as the ‘representation logic’, or they mobilize voters with positive and/or negative statements about social groups, in line with their voters’ feelings towards said groups – we call this the ‘reference logic’.

We combine two data sources to test our arguments: (1) an original dataset of party appeals based on a content analysis of election material in the context of recent elections in Germany (2009, 2013), Switzerland (2011, 2015) and the Netherlands (2012, 2017) and (2) existing survey data that measures party choice and attitudes towards various social groups among voters.

This study makes three contributions. First, it is part of a small yet growing number of studies that emphasize that parties mobilize voters not only through policies but also through group appeals (Damhuis and Karremans, 2017; Evans and Tilley, 2017; Horn et al., 2021; Huber, 2021; Stuckelberger, 2019; Thau, 2019). Second, and in contrast to most of the studies on group appeals, it also highlights the importance of negative statements aimed at groups such as employers or immigrants. It also links data on group appeals with survey data and engages directly with the cleavage and realignment literature, offering a strong theoretical framework on the incentives behind group appeals. Third, most earlier studies on group appeals have taken the form of longitudinal country studies limited to economic groups and focusing on one or two parties, mostly social democrats (Damhuis and Karremans, 2017; Evans and Tilley, 2017; Thau, 2019). By contrast, we offer a comparative and cross-sectional perspective that takes several parties and IP groups into account (see also Horn et al., 2021).

Although our arguments are rooted in a bottom-up perspective – whereby parties respond to voting behaviour and attitudes towards groups – we acknowledge that parties can also act as political entrepreneurs who politicize or underplay social divisions strategically and actively construct and shift group boundaries (for this argument, see, for example, Enyedi, 2005). Hence, their group-based appeals likely also play a role in shaping voter behaviour and forging the attitudes of voters. Unfortunately, an extensive examination of these bi-directional processes is beyond the scope of this article. Rather, we aim to explain the group appeals parties put forward at a specific moment in time, which warrants our treatment of the relatively stable sociological make-up and attitudes of voters as exogenous factors.

## Continuity and Change in Party–Group Relations

Economic groups, defined in terms of the occupation or resources of their members (like income or education), have played a central role in the structuring of party systems and party competition in Western Europe.^1^ As Lipset and Rokkan (1967) famously argued, the cleavages that gave rise to the main party families have been frozen and the relationship between parties and their original constituencies has been maintained even in the second half of the twentieth century, with the labour-capital cleavage originating in the industrial revolution having the most impact on party competition (Lipset and Rokkan, 1967). Simply put, politics in Western Europe has been structured along an economic left-right conflict. The left, backed by workers and lower income groups, favours more state intervention and redistribution, whereas a liberal right, supported by employers and higher income groups, advocates more market liberalism (Goldberg and Sciarini, 2014; Kriesi et al., 2008).

Researchers generally agree that the economic cleavage has become more pacified since the 1960s. Explanations are more diversified and stress several structural transformations such as the rising of living standards and the correlated rise in post-materialist values (Inglehart, 1990), in addition to the ideological move of social democratic parties to the centre, thereby lessening the importance of their working-class base and the economic conflict (Evans and de Graaf, 2012; Kitschelt, 1994). Nevertheless, the left’s slide towards the centre has not been uniform, nor has it led to a broader convergence of economic positions (Evans and de Graaf, 2012). Particularly on the right, the economic conflict remains structural, backed by groups such as small and large employers and farmers who still show support for the centre right (Oesch and Rennwald, 2018; Walter et al., 2014).

Whereas the class cleavage is in decline, a new cultural conflict has become a structuring force in Western European politics and has reshaped party–group relations. This cultural conflict is, on one hand, about the identity politics of the left, that is, the recognition and protection of women and sexual and ethnic minorities, and, on the other hand, about the identity politics of the right, that is, national sovereignty, EU integration and immigration (Bornschier, 2015; Kriesi et al., 2008, 2012). These new topics have contributed to, and been nourished by, the rise of Green parties and the radical right (Kriesi et al., 2008, 2012). The implications for party–group relations are twofold. First, the new cultural conflict gave rise to a realignment between certain economic groups and parties: workers, people on low incomes and those with lower education, all more conservative culturally, have increasingly voted for the radical right, while the well-educated and socio-cultural professionals have increasingly voted for parties on the left, including social democrats (Kriesi et al., 2008, 2012; Oesch and Rennwald, 2010, 2018; Stubager, 2013). Second, the cultural divide has politicized certain non-economic groups at the centre of identity politics and created (new) alignments between those groups and party families. The literature on women, LGBT people and immigrants shows that these groups are likely to vote for parties on the progressive left that defend their interests (e.g. Inglehart and Norris, 2000; Schaffner and Senic, 2006; Strijbis, 2014). These overall trends are shaped by national contexts and electoral systems, the salience of the class and cultural conflict, or party competition (Kriesi et al., 2008). Hence, three types of aligned groups are central in the contemporary two-dimensional conflict structure: the *traditionally aligned economic groups*, including employers, who continue to vote for their traditional economic party ally; the *realigned economic groups*, including workers and socio-cultural professionals, who have become aligned to a new party family in the wake of the cultural conflict; and the *identity politics groups*, which have become politicized and have aligned with parties on their side of the new cultural conflict.

## Group Appeals

This change in alignments raises the question of the behaviour of political parties. The cleavage literature has analysed the changing voter–party relationships exclusively in terms of policies (Evans and de Graaf, 2012; Kitschelt, 1994; Kriesi et al., 2008, 2012). While recognizing that group consciousness is a necessary condition for a cleavage (Bartolini and Mair, 1990), this research has rarely discussed group consciousness as something parties can tap into independently from policies. This overlooks the fact that groups are not just the target of messages, but often also the message itself.

In addition to policy appeals, parties use group-based appeals to win over members of a group (Holman et al., 2015; Robison et al., 2021). Such group-based appeals take different forms: parties turn the group into the message by nominating candidates from said group (Campbell et al., 1960), by relying on endorsements from organizations close to a group, like unions (Campbell et al., 1960; Mcdermott, 2006) or, most explicitly, by talking about a group. Our interest here is in *group appeals*, defined as the explicitly stated support (positive group appeals) or criticism of group categories (negative group appeals) by parties or candidates.

Group appeals have been the subject of growing interest. They have been analysed in several studies on representation as ‘representative claims’ (Damhuis and Karremans, 2017; Saward, 2010) and in a US-focused literature pertaining to ‘identity appeals’ in the case of groups like Latinos or women, for example (Holman et al., 2015; Valenzuela and Michelson, 2016). Finally, some studies, like ours, analyse group appeals in the changing Western European context (Damhuis and Karremans, 2017; Evans and Tilley, 2017; Horn et al., 2021; Huber, 2021; Thau, 2019).

In the case of the UK, Evans and Tilley (2017) argue that group appeals have played a crucial role in the weakening of class-based voting. In their analysis, party attention to economic group categories has declined, while the Labour party and the Conservatives have converged in their economic group appeals, referring similarly to poor people and the working class (Evans and Tilley, 2017). The focus of parties today is mainly on ‘class-neutral groups’ such as families, which, according to Evans and Tilley (2017: 126), is ‘a way to avoid supporting or opposing any particular social group’. Although Thau (2019) finds no decline in the importance of economic group categories in the UK, he also observes the increasing use of group categories that everybody agrees on. Comparing the evolution of budget speeches by French social democratic governments, Damhuis and Karremans (2017) find that references to employees, the traditional voter base of social democrats, have strongly decreased, while references to the middle classes have multiplied. They conclude that:

[w]hile in the 1980s the PS presented itself very clearly as an advocate of the interests of the lower social classes, the party profiles itself more as a caretaker of society in general today, wishing not to oppose social groups (Damhuis and Karremans, 2017: 15–16).

Horn et al. (2021) analyse parties’ group appeals related to welfare policy statements in Scandinavian countries from 2009 to 2015. As with the other studies, they find that the ‘left, centre and right parties appeal to broad demographic categories rather than class’ (Horn et al., 2021: 9). These studies on the European context, like most on group appeals more generally, focus exclusively on positive group appeals (for an exception, see, for example, Huber, 2021; Rhodes and Johnson, 2017).

The literature suggests that the changes of the last decades have resulted in party competition that is best characterized by catch-all parties: parties no longer seek to represent different electorates but rather engage in a cross-class appeal, with the aim of avoiding offending any particular group. By contrast, and by taking a closer look at the motivations behind the use of group appeals and extending the analysis to include IP group categories and negative group appeals, we argue that parties still have important incentives to diverge in their group appeals.

## Expectations

The motivation for parties to use group appeals lies in the ability to influence voting behaviour, an effect observed by some recent studies (Holman et al., 2015; Robison et al., 2021; Thau, 2021). This influence stems from the two different ways in which voters relate to groups. The first, which has been convincingly described by the social identity theory, is the identification with groups that voters belong to (Tajfel and Turner, 1986). A great number of studies show that identities such as gender, ethnicity or partisanship can affect political behaviour (Bergh and Bjorklund, 2011; Campbell et al., 1960; Lewis-Beck et al., 2008). The second, which has been described by the lesser known reference group theory, is the positive and negative feelings voters can hold towards groups, regardless of whether they are members of them (Miller et al., 1991; Wlezien and Miller, 1997). This can also have an impact on political behaviour, as Wlezien and Miller argue:

if people like certain groups and they perceive those groups as aligned with a particular candidate or party, they should evaluate the politician and party more positively. Similarly, people who dislike the groups should evaluate the particular party and candidate more negatively (Wlezien and Miller, 1997: 629).

These two dimensions of group influence help us identify two factors that structure the group appeals of parties.

First, parties can use group appeals to show members of a social group that their identity and interests are represented, which points to the electoral weight of social groups as a factor. Such group appeals follow a ‘representation logic’, whereby parties use group appeals as a signal of representation to voters, showing that they are on their group’s side. Following the classic cleavage idea, the representation logic should lead to a differentiation of group appeals between parties that represent the groups on either side of a conflict. Accordingly, parties with a higher vote share among a group should support it more often in group appeals.

Second, parties use group appeals not only to tell members of a group they are on their side but also to signal to voters that they share their attitudes towards social groups. Parties can use group appeals to show potential voters that they support their favoured and criticize their most disliked groups. Such group appeals follow a ‘reference logic’, which provides the rationale for a party’s use of negative group appeals. While the representation logic can help us understand the potential costs related to attacking particular constituencies (parties do not want to anger their voters), the reference logic helps us understand the benefits provided by connecting with the negative attitudes voters express towards certain groups.

Let us first discuss our expectations with regard to the representation logic. Where the traditionally aligned economic groups are concerned, we expect the representation logic to be of importance. For these groups, economic identity is crucial in voting for their traditional party ally. For instance, when it comes to the traditional alignment between employers and their historic allies, Oesch and Rennwald (2018: 18) have concluded that ‘the endorsement of the centre-right by employers and their agents is primarily motivated by economic attitudes’.^2^ In line with the representation logic, parties therefore have good reasons to remind their traditional constituencies that they still support them.

H1a. Parties that receive more votes from traditionally aligned economic groups reference them more often in positive group appeals.

For realigned economic groups, we contend that the representation logic is unlikely to matter. The reason is simple: unlike the traditionally aligned groups, realigned groups do not cast their vote based on economic attitudes, but make their choice based on their stance on the new cultural conflict. Workers provide an important example: once a core electorate of social democrats, they have since realigned with the radical right because of preferences on the cultural dimension, notably their opposition to immigration, and this despite their preferences on economic issues and their economic status (Bornschier and Kriesi, 2014; Oesch and Rennwald, 2018). The implications for parties are clear: radical right parties have little to gain by stating support for workers as an occupational group, because their popularity among them is not linked to economic considerations.

The same logic applies to socio-cultural professionals and the highly educated that have become aligned with the left. Those groups are the most distinct in their preferences with regard to cultural rather than economic positions, providing a counter-point to workers on questions such as immigration or gender equality (Kitschelt and Rehm, 2014; Kriesi et al., 2008, 2012) which leads Häusermann et al. (2012: 228) to argue that ‘the shift of [. . .] middle-class voters to the left is driven by cultural, rather than economic factors’. Although other researchers suggest that economic preferences are not wholly irrelevant in shaping the vote of socio-cultural professionals (Oesch and Rennwald, 2018), we posit that parties have limited incentives to tap into their economic identities and expect the following:

H1b. Parties that receive more votes from realigned economic groups do *not* reference them more often in positive group appeals.

For IP groups, that is, women, LGBT people and immigrants, the expectation is the same as for traditionally aligned economic groups. We also expect that the voting behaviour of IP groups matters, that is to say that the representation logic applies. Literature on these groups indicates that they show stable alignments and that the cultural dimension and hence their group identities are relevant for their voting behaviour. Indeed, an important driver behind the alignment of women with left-wing parties (the modern gender gap) seems to be their shared positions on gender equality and women’s identification with their gender (Inglehart and Norris, 2000, 2003; Lewis-Beck et al., 2008). Similarly, while socio-economic characteristics might also have a bearing, the migration background itself seems to affect the voting decision of immigrants in favour of left-wing parties who appear to defend their interests (Bergh and Bjorklund, 2011; Strijbis, 2014). This overall trend sees exceptions for particular groups, as in Germany, where Russian-Germans have traditionally supported the Christian democrats and increasingly also support the radical right (Goerres et al., 2020). Finally, the literature has also identified a ‘sexual identity gap’ (Schaffner and Senic, 2006) showing that gays and lesbians favour left-wing parties indicating that this highly politicized identity is also relevant for the voting behaviour of LGBT people (Turnbull-Dugarte, 2020), which leads us to our next expectation:

H1c. Parties that receive more votes from identity politics groups reference them more often in positive group appeals.

We argue that group appeals are not only influenced by the voting behaviour of groups (the representation logic) but also by voters’ attitudes towards groups to which they do not belong (the reference logic). Groups such as immigrants or employers can evoke strong feelings that often differ along party lines. We therefore expect parties to tap into those attitudes and support groups their voters like while criticizing those voters dislike.

H2. Parties whose voters hold more positive (negative) attitudes towards groups reference them more (less) often in positive group appeals and less (more) often in negative group appeals.

## Data

The empirical analysis is based on data from recent elections in Switzerland (2011, 2015), Germany (2009, 2013) and the Netherlands (2012, 2017). These three countries were selected for two reasons. On one hand, all three have experienced a dampening of the class conflict as well as the rise of identity politics, but to different extents (Kriesi et al., 2012: 25). Switzerland witnessed a comparatively early and profound transformation, characterized by a very successful radical right and the loss by Social Democrats of their working-class support more than elsewhere, in favour of a ‘new left’ identity. In Germany, the changes are less pronounced, with a younger and weaker radical right and a left that still has a stronger working-class base. The Dutch case lies in between these two extremes. On the other hand, the main Western European party families (Christian Democrats, Liberals, Social Democrats, Greens and radical right parties) are present in all three countries. In Germany, we also include The Left (*Die Linke*), as it competes with the Social Democrats for the support of workers and people on low income.

To test our theoretical argument that posits that the electoral behaviour of social groups and attitudes of voters towards them provide the underlying motivations for group appeals of parties, we need to combine two data sources: (1) data on party communication to measure group appeals and (2) data on voters’ behaviour and attitudes.

To measure group appeals in party communication, we analyse three party-controlled communication channels: party manifestos, press releases and TV ads (newspaper ads in the Swiss case). These channels are often used to understand party messaging, and their different nature and audiences allow us to gain a comprehensive picture of group appeals (for additional information on the data corpus, see Online Appendix B). Data collection is based on a relational content analysis, inspired by the core sentence approach (Dolezal et al., 2014; Kriesi et al., 2008), and seven human coders, including the authors, were responsible for the coding. The coding unit is the core sentence, which in most cases corresponds to the natural sentence, except for the occurrence of multiple political issues or political actors, in which case each of them constitutes a separate core sentence. We coded up to three group appeals per core sentence, a limit which was only rarely maxed out (<5% of sentences including group appeals). Appeals to groups are coded if a party (1) utters direct criticism or support of a group (e.g. ‘Our party supports workers’), (2) expresses appraisal of a group (e.g. ‘Doctors are important’), (3) emphasizes the effects of a policy on a specific group (e.g. ‘Collective labour agreements protect employees’) or (4) if parties reserve a policy for a certain group (‘We should reduce taxes for businesses’).

We assessed intercoder reliability between the members of the research team (in German and French), on one hand, and, on the other hand, between the student coders (in Dutch). Our variable of interest, the occurrence of group appeals in sentences, is a relatively rare event. With such a skewed distribution, the standard measure of intercoder reliability (Krippendorff’s Alpha) provides too conservative a measure, overestimating the chance agreement between coders (Lacy et al., 2015; Quarfoot and Levine, 2016). For this reason, we report the results for Gwet’s AC1 measure, which together with the Brennan–Prediger index (returns almost identical results for our data) has been suggested as a solution (Lacy et al., 2015; Quarfoot and Levine, 2016) (for further indices including K α, see Online Appendix A). For the Swiss and German data, AC1 is 0.92 (confidence interval (CI): 0.89–0.95), and for the Dutch data, it is 0.76 (CI: 0.71–0.81).^3^ While the lower agreement for the Dutch data requires a cautious interpretation of the data, we trust that thanks to post-coding treatment we reached acceptable data quality. In a post-coding step, we relied on the string variable, which we had used to record each specific group reference as it appeared in the original text, to identify and correct obvious and repeated misclassifications. It also allowed us to sort group categories into groups, such as socio-cultural professionals, that were not part of the original coding scheme (see Online Appendix C for more details).

## Operationalization of Dependent Variables

Based on our theory and the varying expectations with regard to the representation logic, we are interested in positive group appeals to traditionally aligned economic group categories, realigned economic group categories and IP group categories. Regarding our expectations of the reference logic, we are additionally interested in group categories that are used in negative group appeals. Table 1 provides an overview of the individual group categories analysed for those different types. They were selected based on the groups the literature puts an emphasis on and based on the salience of group categories in our data. The economic groups discussed in the cleavage literature are predominantly occupational groups, but also include income and educational groups (e.g. Kriesi et al., 2008, 2012). Our selection of occupational categories is inspired by Oesch’s (2006, 2008) class scheme, which helps make sense of the class support demonstrated by parties in advanced post-industrial societies. We simplified and adapted the Oesch (2006, 2008) class scheme, because in addition to a voter logic, whereby individuals within a general category behave similarly (e.g. professions summarized under the header of socio-cultural professionals), a communication logic must be taken into account: parties use categories that are summarized under a common category in a similar way.

**Table 1.** Analysed Group Appeals.

Type	Group category	Positive appeals	Negative appeals
Traditionally aligned	Large employers	x	x
	Small employers	x	
	Employees	x	
	Farmers	x	
	Rich people		x
Realigned	Workers	x	
	Poor people	x	
	Socio-cultural professionals	x	
	Students	x	
IP	Women	x	
	LGBT people	x	
	Immigrants	x	x

IP: identity politics; LGBT: lesbian, gay, bisexual and transgender.

To study positive group appeals to traditionally aligned economic group categories, we include large and small employers, farmers and employees. Large and small employers are included because they still show disproportionate support for centre-right parties (Oesch and Rennwald, 2018). Diverging from Oesch (2006, 2008), we include managers in the category of large employers: in terms of public perception (both are often criticized by left-wing parties) and political behaviour, managers are very similar to large employers (Oesch and Rennwald, 2018). Farmers also seem to still support their traditional allies, which in Germany and the Netherlands are the Christian democratic parties and the former agrarian radical right party in Switzerland (Knutsen, 2006; Rennwald, 2014; Walter et al., 2014). We also include the umbrella category of ‘employees’, which does not summarize certain professional groups but captures explicit references to the general employed population. While rarely discussed in the literature on voting behaviour, in political communication this group category is an important one, traditionally used by left-wing parties to reach out to their core constituency (Damhuis and Karremans, 2017; Thau, 2018). This brings us to consider employees as a traditionally aligned group; although the type of employee that provides the strongest support for the left has changed (from workers to the new middle class), the left still enjoys disproportionate support among this substantial group.

Realigned group categories include workers, socio-cultural professionals, poor people and students. Occupational groups of workers and socio-cultural professionals are the most frequently discussed of the realigned groups (Kriesi et al., 2008, 2012; Oesch and Rennwald, 2018). Again diverging from Oesch (2006, 2008), we excluded security professionals (policemen and soldiers) from the category of workers, because they are very different objects in a communication logic, with left-wing parties being quite critical of the security apparatus of the state. Poor people are also considered a realigned group. They are particularly supportive of radical right parties, which is unsurprising given the overlap with the group of workers (Han, 2016). The category of poor people also includes references to the unemployed and to social welfare recipients more generally. In political debates, welfare recipients may be discussed in a more critical tone than poor people generally speaking, which stresses our focus here on positive group appeals. Stubager (2013), among others, has suggested that realignment concerns educational rather than occupational groups. We hence also include higher education students, the only salient educational group category in our data on party communication.

We consider positive group appeals to three IP group categories: women, LGBT people and immigrants. Empirically, references to LGBT groups primarily focus on homosexuals, while the category of immigrants also includes people with migration background, refugees and asylum seekers.

Finally, we also study negative group appeals. In our data, substantial amounts of criticisms are present with regard to three group categories: large employers, rich people and immigrants. While positive and negative references are present for employers and immigrants, the category of rich people is overwhelmingly used in critical statements and will be analysed only in terms of negative appeals. Altogether, we therefore distinguish between 3 types of group appeals, which include 14 group categories – counting positive and negative group appeals to the same group category separately, as they are analysed independently (see Table A11 in Online Appendix C for examples of the terms that were coded as references to each group category).

Our dependent variables measure the number of group appeals voiced by parties during an election referencing these 14 group categories. Our dataset includes a total of 9974 group appeals (Germany = 4849, Switzerland = 1278, Netherlands = 3847). With 15 parties competing in 2 elections and 1 party (AfD) in one of them, this amounts to 31 observations for each individual group category. Given that our hypotheses regarding the representation logic (H1a–H1c) are formulated for positive group appeals to three general types of group categories – traditionally aligned economic group categories, realigned economic group categories, IP group categories – we then aggregate the observations for the specific group categories which belong to the same general type. For example, in the case of traditionally aligned economic group categories, we combine the observations for four group categories (large employers, small employers, farmers, employees), which results in a dataset of 124 observations (4 × 31). The dataset for realigned economic group categories accordingly comprises 124 observations (4 × 31), and there are 93 observations for IP group categories (3 × 31). Given that our hypothesis regarding the reference logic should apply equally to all three types of group categories, we aggregate all observations with attitudinal data to test H2. We do so separately for positive and negative appeals, with datasets of 210 (7 × 30) and 90 (3 × 30), respectively.^4^ We analyse these count variables with negative binomial regressions because the data are over-dispersed, which excludes the alternative of Poisson regressions. Given that the likelihood of group appeals rises in line with the amount of campaign communication a party produces, we include the total number of core sentences as exposure variable.^5^ To deal with the repeated measures for each party, which violates the assumption of independent observations of standard multivariate model, we use clustered standard errors. We add the six country-specific elections as fixed effects to account for contextual influence and control for the specific group categories within each group type, because they display different levels of salience.

## Operationalization of Independent Variables

We have argued that group appeals can be motivated by two logics: a representation logic and a reference logic. The measurement of both logics requires survey data. The *representation logic* is operationalized by the vote share of a given social group among party voters and measured based on survey data from the European Social Survey (ESS) for the last election before our period of study.^6^ The share of respondents belonging to a particular group among party voters is calculated for each party–group pair. This indicator is then standardized on the level of each group such as women (datasets of 31 observations), indicating the over- or underrepresentation of a group among voters of a party expressed in standard deviations. This results in a measure of voting support comparable between groups and further allows us to create a unified independent variable of voting support in a combined dataset on the level of each type of group appeal such as IP group categories (3 × 31 = 93 observations). For occupational groups, we rely on the adapted Oesch scheme to identify the voting groups of interest (for details see Table A2 in Online Appendix A). Income groups are sorted by placing respondents into three groups using a common definition of the middle class that identifies respondents as falling into the interval of 75% to 125% of median household income^7^ (e.g. Pressman, 2007). Poor and rich people are therefore defined as respondents with less than 75% and more than 125% of median household income, respectively. We define students as respondents between 18 and 29 years old, who are currently studying at university (identified as those who indicate education as main activity and who hold a degree that provides access to university) or hold a university degree. The latter group of recent students is included to reach a bigger sample size for this rather small group. LGBT people are operationalized as same-sex partners and immigrants as people of first and second generation.

The *reference* logic is defined by the attitude of a party’s voters towards a particular group. Unfortunately, in the European context, no survey regularly queries voters’ perception of groups, in the way that the feeling thermometer in the American National Election Studies does. As a substitute we rely on various questions that tap into perceptions of groups in the European Values Study from 2008 (European Values Study, 2016) and the ESS (2008). Attitudes towards employees/workers and employers are measured based on the confidence of respondents in unions and major companies, respectively. For attitudes towards poor people and immigrants, we use a question that asks if respondents are concerned with the living conditions of the unemployed and immigrants, respectively (for all the questions, see Online Appendix Table A2). Each question is dichotomized, dividing respondents into those with positive and those with negative attitudes towards the group. The percentage of voters with positive attitudes is then calculated for each party–group pair, before this score is standardized on the level of each group, allowing the creation of a common independent variable of voters’ attitudes towards groups for each type of group appeals studied. Given that we lack data on voters’ attitudes towards some groups (e.g. small employers, socio-cultural professionals), we run our models stepwise (see below).

## Analysis

**Figure 1.**
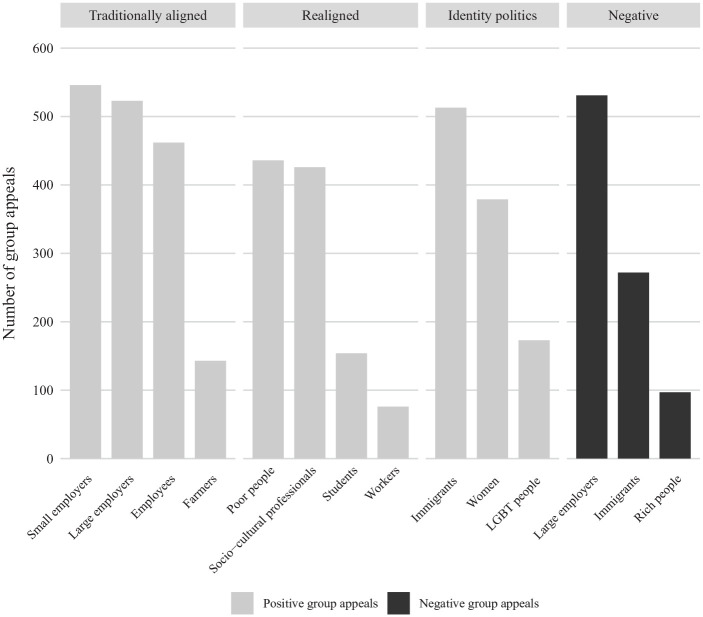
Importance of Group Categories. Note: Data are from Swiss (2011/2015), German (2009/2013) and Dutch (2012/2017) Party Communication. N (Group Appeals): 9974.

Before analysing the drivers behind parties’ group appeals, it is worth looking at their relative importance. Figure 1 displays the frequency of the group categories of interest (see Online Appendix A, Figure 1A for further information on the level of parties). As the total number of group appeals corresponds to around 10,000, a frequency of 100 corresponds to around 1% of all group appeals. Among the economic group categories, positive group appeals to employers, employees, poor people and socio-cultural professionals are all rather salient. The lack of attention for workers – also found in the UK context (Evans and Tilley, 2017; Thau, 2019) – is noteworthy. Historically, workers have been the most politicized occupational group and they still constitute the biggest occupational group in Switzerland, Germany and the Netherlands (European Social Survey, 2010, 2012, 2014). Yet party attention today is more strongly focused on socio-cultural professionals, such as teachers and healthcare professionals, and on employees more generally, the latter implicitly including workers of course. Immigrants are the most salient IP group category, while large employers are clearly the group category most frequently criticized.

Recent studies have given the impression that group appeals nowadays largely lack a link to parties’ voter bases – referring mostly to the same broad group categories (Damhuis and Karremans, 2017; Evans and Tilley, 2017; Horn et al., 2021). In the following, we will test our argument that the electoral support parties receive from a social group (except for the case of realigned groups) and their electorate’s attitudes towards social groups are drivers of group appeals. We start by investigating positive group appeals to the traditionally aligned economic group categories, followed by the realigned economic group categories and IP group categories, before moving on to an overall assessment of the influence of voters’ attitudes on positive and negative group appeals.

**Figure 2.**
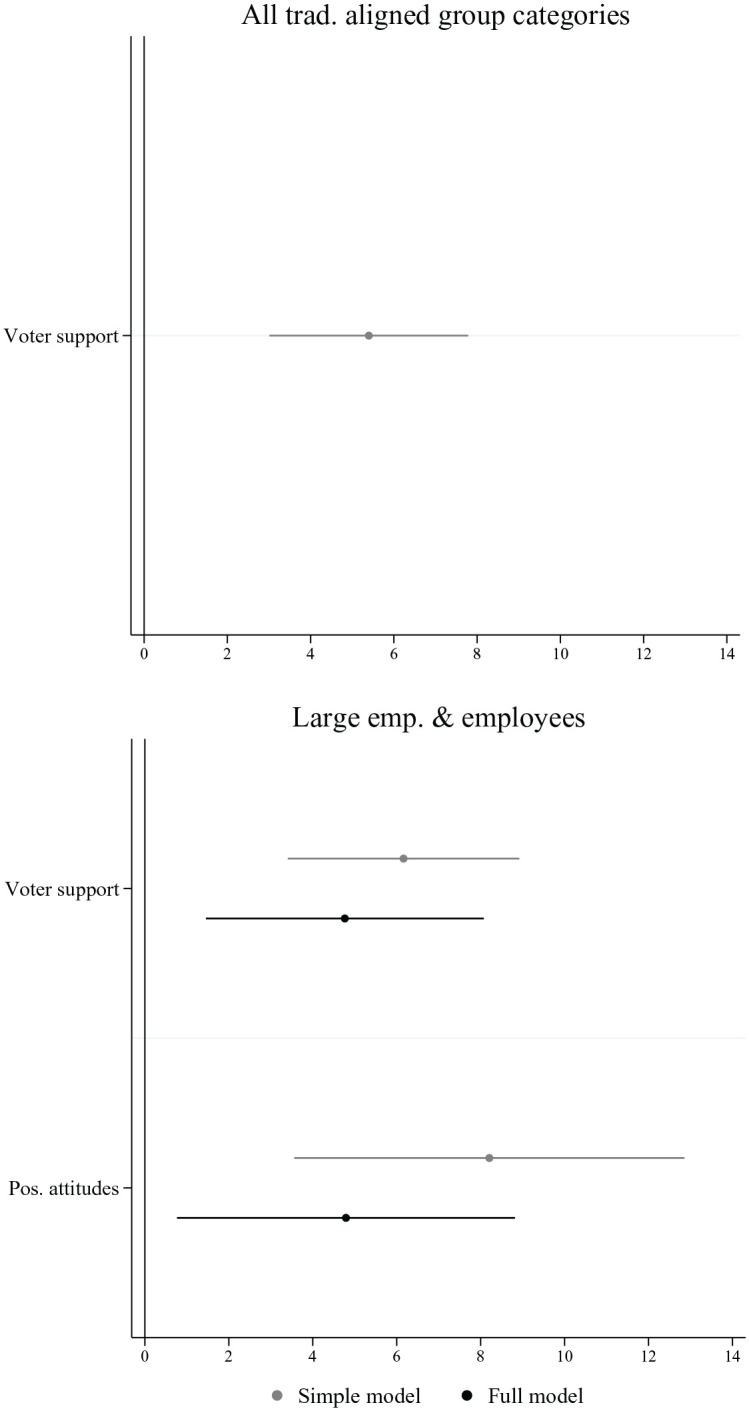
Appeals to Traditionally Aligned Economic Group Categories. Note: Figure displays the average marginal effects on the number of group appeals. Results are based on two distinct models for each independent variable (simple model) and a third model, which includes both factors (full model). Country-election and group category fixed effects are included in all models (see regression Table A3 in the Online Appendix).

Based on negative binomial regression models, Figure 2 presents the average marginal effects of voter support among a social group and voters’ attitudes towards it on the number of positive group appeals (see Regression Table A3 in Online Appendix). The first plot shows the results for all traditionally aligned economic group categories, that is, large employers, small employers, farmers and employees. The second plot focuses on large employers and employees, the group categories for which we possess attitudinal data. It displays the results of the full model that includes voting support and group attitudes as well as two simple models in which the two factors are tested separately.

All the models displayed in Figure 2 indicate that parties that depend more heavily on those groups in electoral terms more often reference traditionally aligned group categories in their campaigns, providing support for Hypothesis 1a. The first plot shows that, considering all group categories, a one-unit increase in voting support (min: −2.2, max: 3.2) leads to an increase of around 5 in the number of group appeals per election. Based on the incident rate ratios (reported in Regression Table in Online Appendix), this effect can also be understood as an increase of 49% in the rate of using these group appeals. In the model that only includes large employers and employees, this substantial effect of voting support remains once we add voters’ attitudes to the model. These results are consistent with the descriptive analysis (see Online Appendix A, Figure 1A), suggesting that the traditional champions still play an important role. Support for small and large employers is provided by liberal parties above all, while traditional left-wing parties support employees, and appeals to farmers come mainly from Christian democrats and from the former agrarian radical right party in Switzerland.

Figure 2 also provides initial supporting evidence for the reference logic. Parties with more voters who share positive attitudes towards large employers or employees more often champion them in group appeals. A one-unit increase in voters with positive attitudes towards those groups (min: −1.6, max: 2) is associated with almost five more positive references to them (full model). In the full model that includes both voter support and voters’ attitudes, the effect of both factors is reduced compared to the simple models. This can be explained by the positive correlation of these factors as members of a group normally hold more positive attitudes towards that group and may be more likely to vote for parties featuring voters who are well disposed towards them.

**Figure 3.**
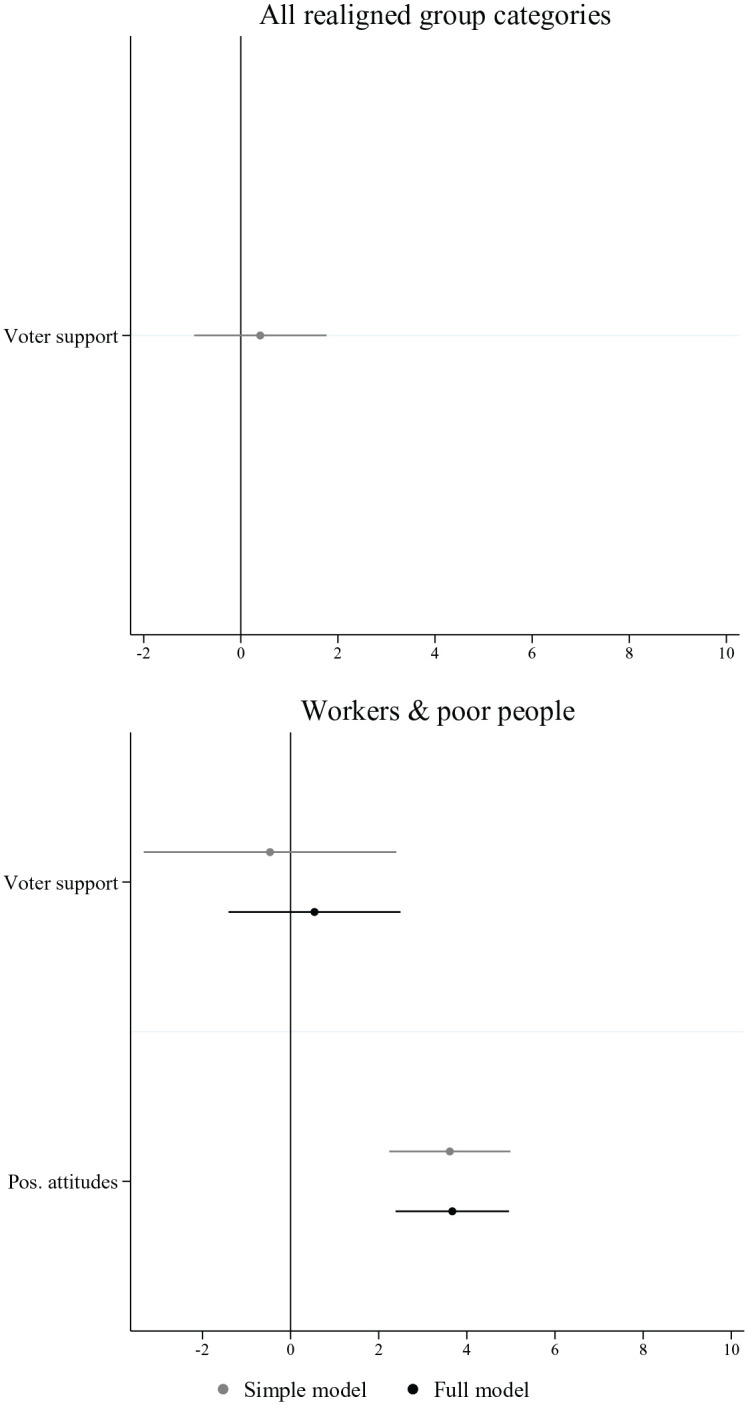
Appeals to Realigned Economic Group Categories. Note: Figure displays the average marginal effects on the number of group appeals. Results are based on two distinct models for each independent variable (simple model) and a third model, which includes both factors (full model). Country-election and group category fixed effects are included in all models (see regression Table A4 in the Online Appendix).

Figure 3 shows the average marginal effects for the realigned economic group categories. They are in line with the expectation that the representation logic is *not* present in those cases (H1b). The graph on the left-hand side indicates that in the model that includes all realigned group categories, that is, workers, poor people, socio-cultural professionals and students, the number of group appeals referencing them does not depend on their electoral support for parties. The descriptive data qualifies this overall assessment somewhat, indicating that some left-wing parties are among the parties most frequently referencing students and socio-cultural professionals (see Online Appendix, Figure 1A). The plot on the right-hand side of Figure 3 only includes the groups for which we have attitudinal data. It similarly shows that when voters’ attitudes towards workers and poor people are added, voter support has no effect on group appeals towards these group categories. However, parties whose voters hold more positive attitudes towards workers and poor people emphasize them more frequently in their election campaigns. Group appeals to workers and poor people therefore provide further support for the reference logic.

**Figure 4.**
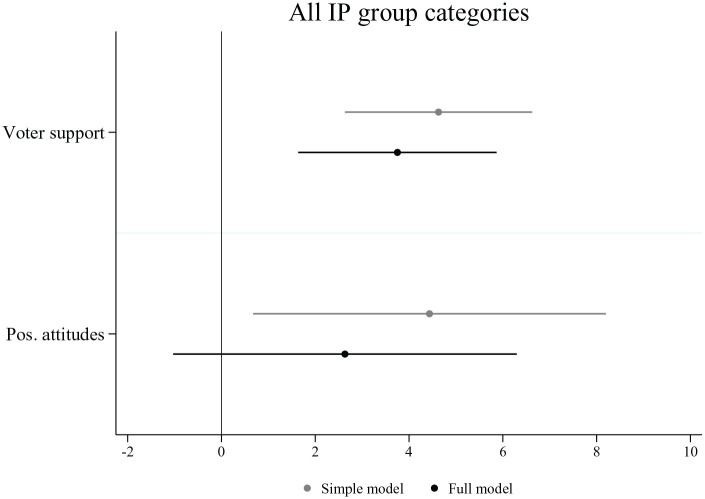
Appeals to IP Group Categories. Note: Figure displays the average marginal effects on the number of group appeals. Results are based on two distinct models for each independent variable (simple model) and a third model, which includes both factors (full model). Country-election and group category fixed effects are included in all models (see regression Table A5 in the Online Appendix).

The results for IP group categories resemble those for traditionally aligned economic group categories. The average marginal effects shown in Figure 4 provide support for H1c and the presence of the representation logic. Parties with more electoral support among IP groups, that is, women, LGBT people and immigrants, refer to them positively more frequently. When adding voters’ attitudes to the model (full model), the effect of voter support remains positive and significant. In the simple model, voters’ positive attitudes towards IP groups also have a positive effect on the number of group appeals. Yet this is no longer the case once voting support from these groups is added to the model. This suggests that the positive effect for positive attitudes is driven by parties that also receive more votes from members of IP groups, who naturally hold more positive attitudes. Therefore, there seem to exist no independent effect of voters’ attitudes on group appeals towards IP group categories. In contrast to the results for traditionally aligned and realigned economic group categories, the results for IP group categories run counter to the expectations of the reference logic (H2).

So far, we have looked at the effect of voters’ attitudes separately for the three types of group categories. Given that H2 applies to all types of group categories and positive as well as negative group appeals, an overall test of the reference logic requires two additional models: a first model, which combines all positive appeals towards group categories for which we have attitudinal data, and a second model, incorporating all negative group appeals, that is, the criticism of large employers, rich people and immigrants.

**Figure 5.**
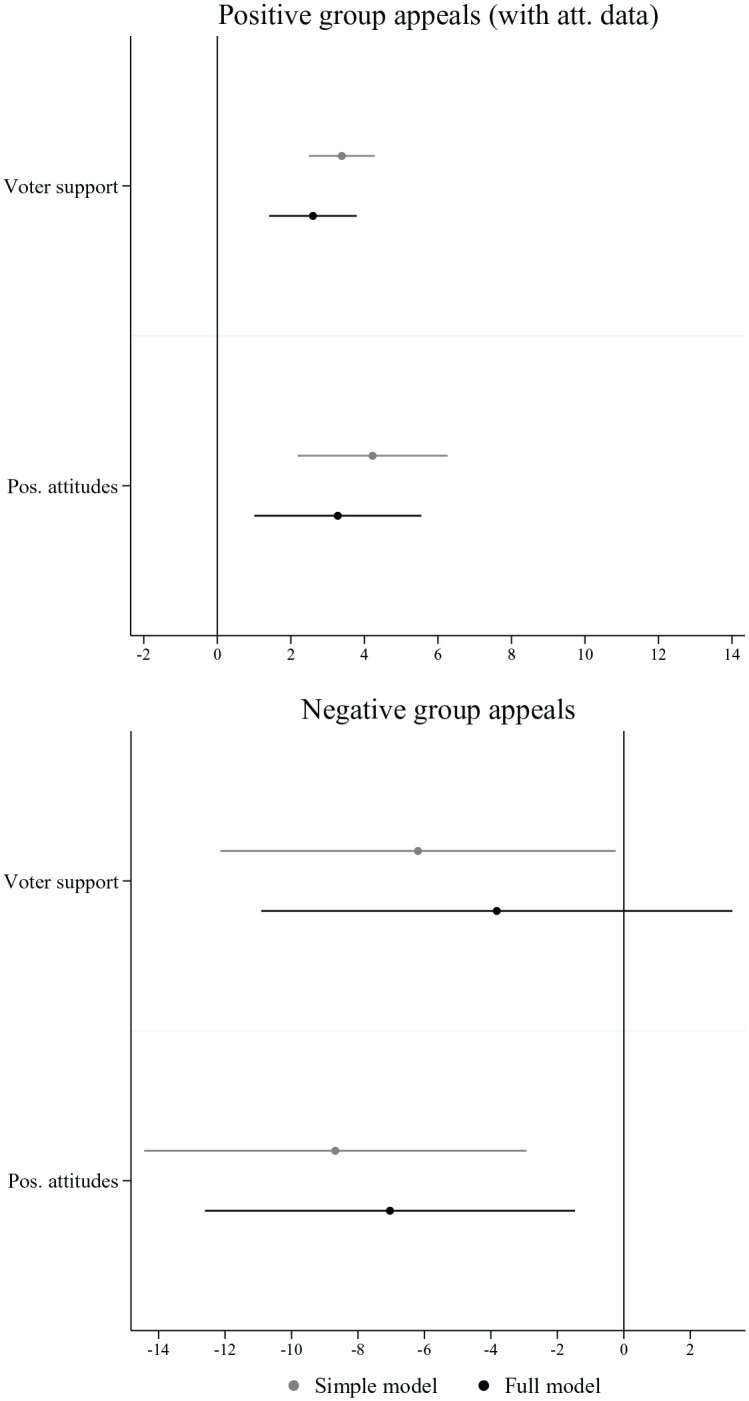
Reference Logic. Note: Figure displays the average marginal effects on the number of group appeals. Results are based on two distinct models for each independent variable (simple model) and a third model, which includes both factors (full model). Country-election and group category fixed effects are included in all models (see regression Table A6 in the Online Appendix).

The results of these two models can be found in Figure 5. Overall, they show that parties with more voters who harbour positive views about particular groups reference them more often in positive group appeals. The second plot also indicates that parties with voters who hold more positive attitudes towards the group criticize it less often in negative group appeals. Conversely, parties with more voters who display negative feelings or who show less concern for large employers, rich people and immigrants criticize them more frequently in group-based messages. Hence, the negative attitudes of voters seem to provide an important motivation of criticism towards group categories, a behaviour that is inconsistent with a representation logic, but in line with the reference logic.

Overall, the results on both positive and negative group appeals suggest that parties’ group appeals are not only driven by the electoral weight of social groups among a party’s electorate but also by those voters’ attitudes towards social groups. The findings, by and large, support H2 and emphasize the importance of the reference logic. Nevertheless, and as the results for IP group categories indicate, this does not mean that the reference logic is always present. For many group appeals we could not test this argument because we do not possess data on voters’ attitudes. This absence can be expected to be linked to a lack of salience of those attitudes in many cases.

## Conclusion

In the 1960s, Lipset (1960: 220) argued that parties’ ‘*appeals* [emphasis added] and their support suggest that they do represent the interests of different classes’. In the meantime, the class conflict has weakened while a cultural conflict has taken centre stage. Some recent studies have suggested that those changes have led parties to pursue catch-all strategies that focus in their group appeals on more class-neutral demographic groups and consider all classes as potential constituencies (Damhuis and Karremans, 2017; Evans and Tilley, 2017; Horn et al., 2021). By contrast, we have argued that parties’ group-based messaging still reflects their electoral support among key social groups. Our findings indicate that for traditionally aligned groups as well as IP groups, parties that enjoy more electoral support among them address them significantly more often with positive group appeals.

We have shown that parties’ group messaging follows a different logic regarding groups that have been realigned in the wake of the cultural conflict. As expected, our findings suggest that the new champions of workers, socio-cultural professionals and students do not reach out to them mainly based on those economic/educational identities.

Group appeals to workers and poor people have traditionally been the most visible illustration of the representation logic and of Lipset’s (1960: 220) view of parties as engaged in the ‘translation of class struggle’. Nowadays, they seem to illustrate the importance of the reference logic and the response of parties to their voters’ solidarity with other groups. Our results show more generally that group appeals should not exclusively be understood as representative claims that aim to represent the group mentioned, but should further be understood as a means to indicate to the electorate towards which groups the party’s solidarity or opposition is directed. This reference logic also helps us make sense of criticism of group categories such as the rich, employers or immigrants.

Focusing on three countries that vary in their stage of cleavage transformation, we can expect that many of our results would be valid in other multiparty Western European countries with a two-dimensional conflict structure. We would expect that deviations from our results would mainly concern group appeals to realigned economic groups. Indeed, it is possible that in contexts where the radical right adopts a more left-leaning and welfare chauvinist stance (see, for example, Otjes et al., 2018), their campaign communication is also more likely to champion workers and the poor. Party systems are known to strongly shape party competition and results should therefore not be generalized to the two-party system. This is particularly true for the UK, where previous studies have found rather different results (Evans and Tilley, 2017; Thau, 2019).

Three limitations of this study should be addressed. The first is that the causal argument could run in the other direction, as briefly discussed in the introduction. Our analysis gives some credit to our bottom-up approach and shows that parties’ group appeals do, to some extent, respond to their voters’ social make-up and attitudes towards social groups. However, future research should also pay attention to the role of party agency and investigate the influence of parties’ group-based messaging on voting behaviour as well as group attitudes. A second limitation is that our approach does not delve into the relationship between group appeals and broader political values and ideologies. The positioning of parties as well as voters towards groups such as immigrants, women and employers are linked to political values such as nationalism, equality and liberalism – the relation between party positioning and such values would therefore be worth investigating. The third limitation is that the operationalization of voters’ attitudes is not ideal as we lacked satisfying standardized survey questions, such as the thermometer questions in the US-American context.

Despite these limitations, this article offers a step towards a better understanding of party–group relations in contemporary Western Europe, emphasizing the continuing links between social groups and parties in terms of attitudes and voting behaviour. This article is part of a growing literature that stresses that party competition is not only about which policies parties are advocating but also for whom or against whom they position themselves. Such research helps to flesh out the supply side of what Achen and Bartels (2016) call the group theory of democracy. To advance research on group appeals in Western Europe, we deem it particularly important to gather more and better data on the perception of social groups and the association of social groups with political parties to study the effects and drivers of group appeals and to understand how they may contribute to the growing affective polarization between political camps as well as between social groups that we currently experience in many Western democracies.

## Supplemental Material

sj-pdf-1-psx-10.1177_00323217221123147 – Supplemental material for Group Appeals of Parties in Times of Economic and Identity Conflicts and Realignment Supplemental material, sj-pdf-1-psx-10.1177_00323217221123147 for Group Appeals of Parties in Times of Economic and Identity Conflicts and Realignment by Simon Stuckelberger and Anke Tresch in Political Studies

## Notes

1. Educational groups are considered as economic groups, because education constitutes an important resource and is strongly linked to occupation and income. The three are often discussed together as socio-economic characteristics, which are ‘related, at least loosely, to “class”’ (Pontusson, 2015: 1).

2. Although economic identities such as ‘employers’ and economic attitudes are not identical, they are connected. With data on the influence of these economic identities lacking, the extent to which economic or cultural attitudes matter for voting choices of economic groups offers an indication for the effect of the respective identities.

3. In 91% of the sentences, all coders agreed on unitizing. The intercoder reliability of the coding step is based on coders who agreed on unitizing.

4. For one party (AfD, Germany) we lack data on voters’ attitudes, which reduces the number of observations to 30.

5. The exposure variable allows count models to take into account that observations may vary in their likelihood of producing events depending on the period of time or area of space. In our example, we count group appeals in different communication materials of varying length.

6. Swiss elections (2011): ESS (2008, 2010), Swiss elections (2015): ESS (2012, 2014); German elections (2009): ESS (2006, 2008), German elections (2013): ESS (2010, 2012); Dutch elections (2012): ESS (2010), Dutch elections (2017): ESS (2012, 2014).

7. The household income is adjusted to the household size by dividing the household income by the square root of the number of people living in the household (for the same adjustment, see Organisation for Economic Co-operation and Development (OECD, 2011)).
