# Supplementary material for: Group Appeals of Parties in Times of Economic and Identity Conflicts and Realignment
Source: Polit Stud (Oxf). 2022 Sep 24;72(2):463–85. doi: 10.1177/00323217221123147 (PMC11070317; doi:10.1177/00323217221123147)
Supplement: sj-pdf-1-psx-10.1177_00323217221123147 – Supplemental material for Group Appeals of Parties in Times of Economic and Identity Conflicts and Realignment [file sj-pdf-1-psx-10.1177_00323217221123147.pdf]

Online Appendix

A) Additional Tables and Figures

Table A1 Indexes of intercoder reliability

	AC1		Brennan-Prediger		Krippendorff α		Percent Agreement	
German/Swiss data	0,922	(0,889-0,955)	0,919	(0,885-0.953)	0,708	(0,607-0.809)	0,923	(0,891-0,955)
Dutch data	0,760	(0,709-0,812)	0,754	(0,701-0,807)	0,460	(0.365-0,555)	0,766	(0,715-0,816)

Table A2 Operationalization of main variables

	Type	Group Categories	Measurement of voter support (ESS)	Measurement of attitudes towards group categories	Considered as support	Other modalities
Economic	Occupation	Workers	Oesch's 16 class categories*: 7, 8, 15 (without security prof.), 16	Confidence in trade unions (EVS 2008)	A lot<br>Quite a lot	Not very much<br>None at all
		Socio-cultural prof.	Oesch's 16 class categories: 13, 14	NA		
		Employees	Oesch's 16 class categories: 5-8, 11-16	Confidence in trade unions (EVS 2008)	A lot<br>Quite a lot	Not very much<br>None at all
		Farmers	Isco 6000-6400 (in Oesch's class schema, part of 3,4,7,8)	NA		
		Small Employers	Oesch's 16 class categories: 2, 3, 4	NA		
		Large Employers	Oesch's 16 class categories: 1, 9, 10	Confidence in major companies (EVS 2008)	A lot<br>Quite a lot	Not very much<br>None at all
	Income	Poor people	< 75 % * median(adj. household income)	Level of concern for living conditions of unemployed? (EVS 2008)	Very Much<br>Much	To a certain extent<br>Not so much<br>Not at all
		Rich People	> 125 % * median(adj. household income)	Disagreement with "People with high salaries should pay a higher share of their earnings in tax." (ESS 2008)	Disagreement	Agreement
	Education	Students	18-29 years old & university/upper high school degree & Main activity last 7 days: in education OR 18-29 years old & university degree	NA		
Identity politics	Gender	Women	Sex	Disagreement with "When jobs are scarce, men should have more right to a job than women." (ESS 2008)	Disagree strongly<br>Disagree	Neither agree nor disagree<br>Agree strongly<br>Agree
	Sexuality	LGBT people	Same-sex partners	don't like as neighbours: homosexuals (EVS 2008)	Not mentioned	Mentioned
	Citizenship	Immigrants	Immigrants of first/second generation	Level of concern for living conditions of immigrants (EVS 2008)	Very Much<br>Much	To a certain extent<br>Not so much<br>Not at all

*Oesch's 16 class schema: 1 Large employers, 2 Self-employed professionals, 3 Small business owners with employees, 4 Small business owners without employees, 5 Technical experts, 6 Technicians, 7 Skilled manual, 8 Low-skilled manual, 9 Higher-grade managers and administrators, 10 Lower-grade managers and administrators, 11 Skilled clerks, 12 Unskilled clerks, 13 Socio-cultural professionals, 14 Socio-cultural semi-professionals, 15 Skilled service, 16 Low-skilled service

Table A3 Regression Table: Traditionally aligned economic group categories

	All<br>Simple Model	Large Emp./Emp.<br>Simple Model	Large Emp./Emp.<br>Simple Model	Large Emp./Emp.<br>Full Model
Voter Support	1.490***	1.494***		1.340**
	(0.111)	(0.122)		(0.136)
Pos. Attitudes			1.650***	1.342*
			(0.208)	(0.163)
Group categories (Ref. Employees)				
Farmers	0.345***			
	(0.092)			
Small employers	1.275			
	(0.227)			
Large employers	1.376	1.329	1.394	1.353
	(0.266)	(0.245)	(0.256)	(0.220)
Election (Ref: 1. Dutch election)				
2. Dutch Election	0.975	1.172	1.094	1.157
	(0.201)	(0.221)	(0.244)	(0.237)
1. Swiss Election	0.917	1.308	1.426	1.375
	(0.303)	(0.460)	(0.487)	(0.471)
2. Swiss Election	0.759	1.358	1.389	1.423
	(0.133)	(0.339)	(0.327)	(0.337)
1. German Election	1.113	1.362	2.500***	2.008**
	(0.186)	(0.266)	(0.636)	(0.481)
2. German election	0.762	1.098	2.267**	1.744*
	(0.173)	(0.237)	(0.657)	(0.476)
Constant	0.007***	0.005***	0.004***	0.004***
	(0.001)	(0.001)	(0.001)	(0.001)
Over-dispersion paramenter	0.400***	0.188***	0.210***	0.139***
	(0.083)	(0.050)	(0.071)	(0.039)
Observations	124	62	60	60

Note: The dependent variable is the number of group appeals to one of the traditionally aligned group categories (large employers, small employers, farmers, employees) or to one of them with attitudinal data (large employers, employees). Cell entries are exponentiated regression coefficients (Incident Rate Ratios) from negative binomial regressions with robust standard errors (clustered by party) in parentheses. The number of core sentences is included as exposure variable. In models that include voter's attitudes, the number of observations is reduced because those data are not available for the German Radical Right. *** p<0.001, ** p<0.01, * p<0.05

Table A4 Regression Table: Realigned economic group categories

	All<br>Simple Model	Workers/Poor People<br>Simple Model	Workers/Poor People<br>Simple Model	Workers/Poor People<br>Full Model
Voter Support	1.049	0.944		1.066
	(0.090)	(0.164)		(0.128)
Pos. Attitudes			1.527***	1.539***
			(0.109)	(0.105)
Group categories (Ref. Poor people)				
Socio-cultural prof.	0.843			
	(0.192)			
Students	0.305***			
	(0.080)			
Workers	0.149***	0.157***	0.143***	0.145***
	(0.032)	(0.033)	(0.033)	(0.032)
Election (Ref: 1. Dutch election)				
2. Dutch Election	0.916	0.933	0.942	0.940
	(0.115)	(0.235)	(0.192)	(0.189)
1. Swiss Election	0.195***	0.383	0.231***	0.240**
	(0.092)	(0.225)	(0.101)	(0.107)
2. Swiss Election	0.289***	0.379*	0.250***	0.248***
	(0.101)	(0.154)	(0.084)	(0.084)
1. German Election	0.867	1.325	1.063	1.031
	(0.228)	(0.483)	(0.287)	(0.315)
2. German election	0.537*	0.731	0.612	0.607
	(0.139)	(0.300)	(0.205)	(0.215)
Constant	0.010***	0.008***	0.010***	0.010***
	(0.003)	(0.003)	(0.002)	(0.002)
Over-dispersion paramenter	0.398***	0.422**	0.281**	0.272**
	(0.106)	(0.137)	(0.108)	(0.114)
Observations	124	62	60	60

Note: The dependent variable is the number of group appeals to one of the realigned group categories (workers, poor people, socio-cultural prof., students) or to one of them with attitudinal data (workers, poor people). Cell entries are exponentiated regression coefficients (Incident Rate Ratios) from negative binomial regressions with robust standard errors (clustered by party) in parentheses. The number of core sentences is included as exposure variable. In models that include voter's attitudes, the number of observations is reduced because those data are not available for the German Radical Right. *** p<0.001, ** p<0.01, * p<0.05

Table A5 Regression Table: IP group categories

	Simple Model	Simple Model	Full Model
Voter Support	1.500***		1.360***
	(0.149)		(0.127)
Pos. Attitudes		1.432*	1.241
		(0.212)	(0.187)
Group categories (Ref. Immigrants)			
LGBT people	0.268***	0.289***	0.272***
	(0.051)	(0.060)	(0.053)
Women	0.589*	0.600	0.593
	(0.154)	(0.175)	(0.181)
Election (Ref: 1. Dutch election)			
2. Dutch Election	1.131	1.035	1.102
	(0.255)	(0.282)	(0.247)
1. Swiss Election	0.248**	0.261*	0.232***
	(0.107)	(0.138)	(0.102)
2. Swiss Election	1.697	1.345	1.466
	(0.709)	(0.568)	(0.598)
1. German Election	1.557	1.492	1.587
	(0.536)	(0.502)	(0.491)
2. German election	1.543	1.638	1.667
	(0.467)	(0.533)	(0.451)
Constant	0.007***	0.007***	0.007***
	(0.002)	(0.002)	(0.002)
Over-dispersion paramenter	0.515*	0.572*	0.496**
	(0.138)	(0.134)	(0.120)
Observations	93	90	90

Note: The dependent variable is the number of group appeals to one of the IP group categories (immigrants, LGBT people, women). Cell entries are exponentiated regression coefficients (Incident Rate Ratios) from negative binomial regressions with robust standard errors (clustered by party) in parentheses. The number of core sentences is included as exposure variable. In models that include voter's attitudes, the number of observations is reduced because those data are not available for the German Radical Right. *** p<0.001, ** p<0.01, * p<0.05

Table A6 Regression Tables: Reference logic

	Positive group appeals Simple Model	Positive group appeals Simple Model	Positive group appeals Full Model
Voter Support	1.326***		1.235***
	(0.059)		(0.063)
Pos. Attitudes		1.403***	1.305**
		(0.117)	(0.121)
Group categories (Ref. Employees)			
Large employers	1.408	1.461	1.423*
	(0.273)	(0.296)	(0.241)
Immigrants	1.178	1.204	1.198
	(0.226)	(0.312)	(0.283)
Poor People	0.942	0.945	0.962
	(0.198)	(0.254)	(0.241)
LGBT people	0.351***	0.372***	0.358***
	(0.054)	(0.078)	(0.063)
Women	0.744	0.748	0.751
	(0.135)	(0.140)	(0.146)
Workers	0.167***	0.144***	0.157***
	(0.049)	(0.039)	(0.046)
Election (Ref: 1. Dutch election)			
2. Dutch Election	1.043	1.017	1.043
	(0.179)	(0.170)	(0.163)
1. Swiss Election	0.634	0.530*	0.557*
	(0.197)	(0.145)	(0.157)
2. Swiss Election	1.155	0.965	1.005
	(0.385)	(0.270)	(0.281)
1. German Election	1.350	1.491	1.480
	(0.395)	(0.383)	(0.367)
2. German election	1.211	1.327	1.317
	(0.341)	(0.331)	(0.314)
Constant	0.006***	0.006***	0.006***
	(0.002)	(0.002)	(0.001)
Over-dispersion paramenter	0.440***	0.453***	0.398***
	(0.102)	(0.080)	(0.082)
Observations	210	210	210

Note: The dependent variable is the number of positive group appeals to one of the following group categories: employees, large employers, immigrants, poor people, LGBT people, women, workers. Cell entries are exponentiated regression coefficients (Incident Rate Ratios) from negative binomial regressions with robust standard errors (clustered by party) in parentheses. The number of core sentences is included as exposure variable. In models that include voter's attitudes, the number of observations is reduced because those data are not available for the German Radical Right.
*** p<0.001, ** p<0.01, * p<0.05

	Negative group appeals Simple Model	Negative group appeals Simple Model	Negative group appeals Full Model
Voter Support	0.525*		0.702
	(0.137)		(0.215)
Pos. Attitudes		0.441***	0.521**
		(0.088)	(0.127)
Group categories (Ref. Large emp.)			
Rich people	0.194***	0.238*	0.212**
	(0.075)	(0.141)	(0.120)
Immigrants	0.437	0.410	0.376
	(0.322)	(0.309)	(0.279)
Election (Ref: 1. Dutch election)			
2. Dutch Election	0.902	1.423	1.099
	(0.309)	(0.258)	(0.386)
1. Swiss Election	3.633*	3.232*	3.701**
	-1.873	-1.582	-1.853
2. Swiss Election	2.261*	2.931**	2.956**
	(0.915)	-1.129	-1.219
1. German Election	0.420	0.522	0.430
	(0.260)	(0.293)	(0.253)
2. German election	0.731	0.837	0.734
	(0.409)	(0.468)	(0.418)
Constant	0.008***	0.006***	0.007***
	(0.004)	(0.002)	(0.003)
Over-dispersion paramenter	2.282**	2.260**	2.211**
	(0.587)	(0.665)	(0.655)
Observations	93	90	90

Note: The dependent variable is the number of negative group appeals to one of the following group categories: large employers, rich people, immigrants. Cell entries are exponentiated regression coefficients (Incident Rate Ratios) from negative binomial regressions with robust standard errors (clustered by party) in parentheses. The number of core sentences is included as exposure variable. In models that include voter's attitudes, the number of observations is reduced because those data are not available for the German Radical Right. *** p<0.001, ** p<0.01, * p<0.05

Figure A1 Descriptive figure on group appeals

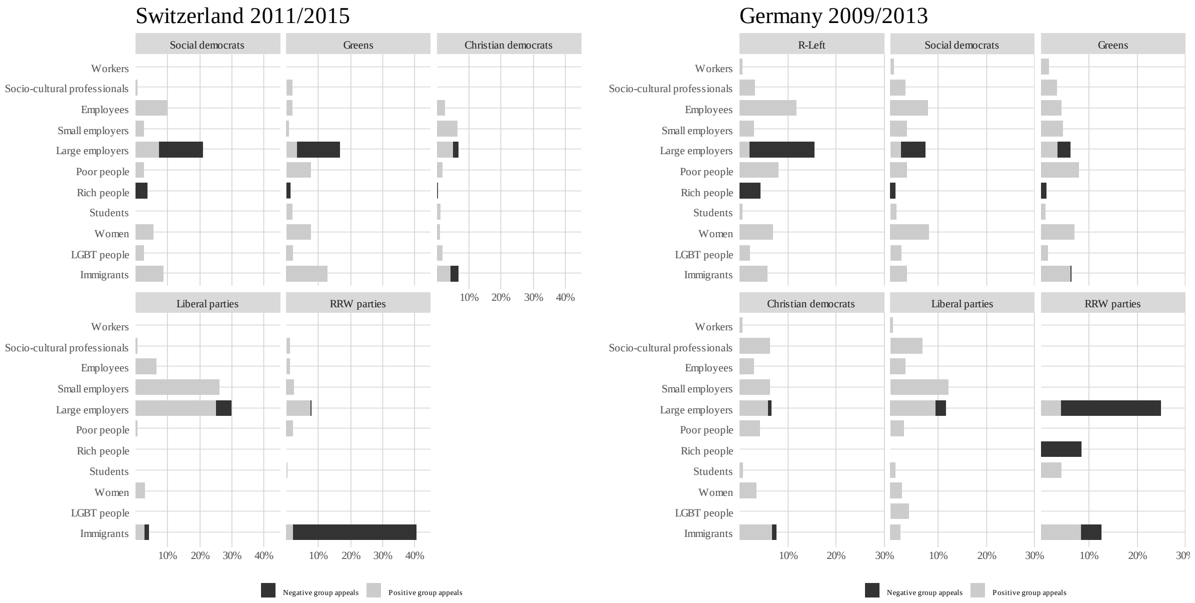

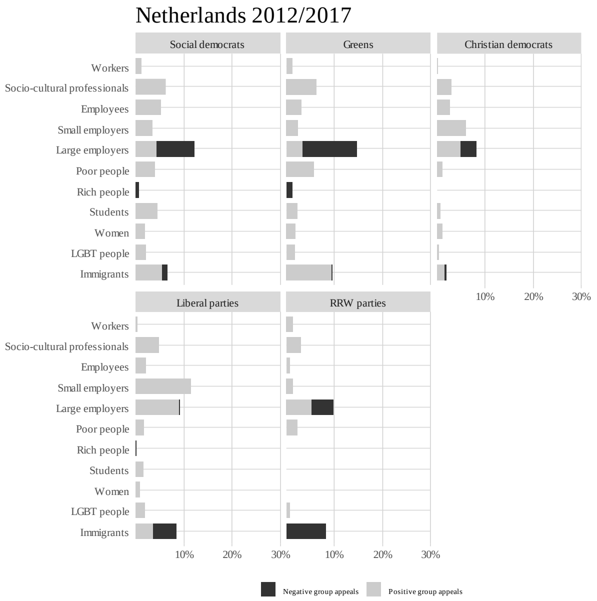

Note: The figure displays the share of group appeals dedicated by parties to group categories. Data is from Swiss (2011/2015), German (2009/2013) and Dutch (2012/2017) party communication.

B) Data corpus

This section of the appendix provides additional information on the corpus we used for the analysis of group appeals. The content analysis is based on three sources: party manifestos, press releases and TV ads. In the Swiss case, political advertising is forbidden on television and radio, which is why we use newspaper advertisements for this country. These sources have different purposes and audiences and therefore allow for a comprehensive overview of party communication. Tables A7, A8 and A9 provide information about the collected data for these various communication channels.

Table A7 Length of party manifestos (number of pages)

	Switzerland		Germany		Netherlands	
Party Families	2011	2015	2009	2013	2012	2017
Radical Left	-	-	64	100	-	-
Social Democrats	12	12	94	120	77	67
Greens	24	25	115	337	38	78
Christian Democrats	16	24	94	128	86	105
Liberal parties	20	3	86	104	64	102
Radical Right-wing parties	20	200	-	4	56	1
Mean	18	53	91	132	64	71

Party manifestos, one for each election, are used by all parties as campaign channels in all three countries. Table A7 shows there is some variation in terms of the length of the documents: in Germany and the Netherlands, they are substantially longer than in Switzerland.

Table A8 Number of TV and newspaper advertisements

	Switzerland		Germany		Netherlands	
Party Families	2011	2015	2009	2013	2012	2017
Radical Left	-	-	1	1	-	-
Social Democrats	38	17	1	1	1	1
Greens	12	2	1	2	1	2
Christian Democrats	21	30	4	3	1	2
Liberal parties	40	54	1	1	0	2
Radical Right-wing parties	106	90	-	1	1	2
Mean	43	39	1,6	1,5	0,8	1,8

For the Netherlands and Germany, we analysed the TV ads which parties ran in the state-allocated free time slots on the public TV channels. Coding involved transcribing the audio of the ad and the visual content was left out of the analysis. Each ad was coded only once, irrespective of the number of times it was aired, given that this information was not available. For Switzerland, newspaper ads were collected from three types of newspapers in the German- and French-speaking parts of the country. The selection includes: two quality newspapers (NZZ, Le Temps), two regional newspapers (Tagesanzeiger, Tribune de Genève) and two tabloids (Blick, Le Matin). For newspapers it was possible to code the relative importance of particular ads by coding each ad, even if it already appeared in another newspaper or on another day. This allows for a more accurate description of the emphasis political parties put on different messages for the Swiss case.

Table A9 Number of press releases

	Switzerland		Germany		Netherlands	
Party Families	2011	2015	2009	2013	2012	2017
Radical Left	-	-	88	14	-	-
Social Democrats	44	29	116	93	99	50
Greens	34	30	60	16	47	53
Christian Democrats	42	22	57	15	28	36
Liberal parties	30	20	31	5	4	24
Radical Right-wing parties	27	20	0	43	11	1
Mean	35	24	70	31	38	33

Press releases were collected for a period of two months prior to the election date. Only press releases with substantial political content are included in the corpus, excluding announcements of upcoming events, for example.

C) Additional information on the coding of group appeals

The original coding of group appeals was based on a variable with 55 groups, which were each listed twice to distinguish between positive and negative references (see Table A10).

Table A10 List of original group categories

Summary Categories	ID	Group Category
Age groups	101	Elderly people (positive)
	102	Elderly people (negative)
	103	Middle-aged people (positive)
	104	Middle-aged people (negative)
	105	Other (positive)
	106	Other (negative)
	107	Young people (negative)
	108	Young people (positive)
Gender/Sexuality groups	301	Heterosexual people (positive)
	302	Heterosexual people (negative)
	303	Homosexual people (negative)
	304	Homosexual people (positive)
	305	Men (positive)
	306	Men (negative)
	307	Other (positive)
	308	Other (negative)
	309	Transgender people (positive)
	310	Transgender people (negative)
	311	Women (positive)
	312	Women (negative)
Family Status groups	401	(traditional) Families (positive)
	402	(traditional) Families (negative)
	403	Alternative forms of families (positive)
	404	Alternative forms of families (negative)
	405	Civil Union (positive)
	406	Civil Union (negative)
	407	Concubinage (positive)
	408	Concubinage (negative)
	409	Married couples (positive)
	410	Married couples (negative)
	411	Other (positive)
	412	Other (negative)
	413	Singles (positive)
	414	Singles (negative)
Citizenship groups	501	Asylum seekers/refugees (positive)
	502	Asylum seekers/refugees (negative)
	503	Foreign nationals/Immigrants (positive)
	504	Foreign nationals/Immigrants (negative)
	505	Naturalized Swiss citizens (positive)
	506	Naturalized Swiss citizens (negative)
	507	Other (positive)
	508	Other (negative)
	509	People with migrant background (positive)
	510	People with migrant background (negative)
Socioeconomic groups	601	Academia (positive)
	602	Academia (negative)
	603	Banks (positive)
	604	Banks (negative)
	605	Blue Collar workers (negative)
	606	Blue Collar workers (positive)
	607	Consumers (positive)
	608	Consumers (negative)
	609	Economic elite (positive)
	610	Economic elite (negative)
	611	Employees (positive)
	612	Employees (negative)
	613	Employers (positive)
	614	Employers (negative)
	615	Farmers (positive)
	616	Farmers (negative)
	617	Firms (positive)
	618	Firms (negative)
	619	Functionaries (positive)
	620	Functionaries (negative)
	621	Homeowners (positive)
	622	Homeowners (negative)
	623	Managers (positive)
	624	Managers (negative)
	627	Other (positive)
	628	Other (negative)
	629	Middle class (positive)
	630	Middle class (negative)
	631	Tax payers (positive)
	632	Tax payers (negative)
	633	Teacher (positive)
	634	Teacher (negative)
	635	Tenants (positive)
	636	Tenants (negative)
	637	The poor (positive)
	638	The poor (negative)
	639	The rich (positive)
	640	The rich (negative)
	641	Welfare recipients (positive)
	642	Welfare recipients (negative)
	643	Criminals (positive)
	644	Criminals (negative)
	645	Victims (positive)
	646	Victims (negative)
	647	Professional groups (positive)
	648	Professional groups (negative)
	649	Unions (positive)
	650	Unions (negative)
	653	Volunteers (positive)
	654	Volutneers (negative)
	655	Journalists (positive)
	656	Journalists (negative)
	657	Religious people (positive)
	658	Religious people (negative)
Political groups	703	Majority (positive)
	704	Majority (negative)
	705	Minority (positive)
	706	Minority (negative)
	707	Non-voters (positive)
	708	Non-voters (negative)
	709	Other (positive)
	710	Other (negative)
	711	Political elite (positive)
	712	Political elite (negative)
	713	Silent majority (positive)
	714	Silent majority (negative)

We also coded a string variable with the actual term used to describe a group. The analysed categories were created based on those two variables (for an overview see Table A11). Some of the categories, like employees, used in this analysis have been coded as such originally. Other categories such as large employers or immigrants are the results of collapsing original categories. Finally, some categories like socio-cultural professionals were created based on the string variable. Figure 11 also indicates the most frequent examples for positive and negative references to each group category.

Table A11 Categorization and examples

Group category	Category creation	Examples (positive appeals)	Examples (negative appeals)
Large Employers	Collapsing of original categories of "firms", "banks",”economic elite", "employers", "managers"	Unternehmen, Betriebe, entreprises, bedrijven, werkgevers,	Unternehmen, Banken, entreprises, bedrijven, banken, multinationals
Small Employers	Based on string variable identifying terms such as "SME" or "entrepreneurs"	Mittelstand, KMU, Unternehmer, PME, ondernemers, starters, MKB	
Farmers	Original category	Landwirte, familles paysannes, (Milch)bauern, boeren, tuinders	
Employees	Original category	Beschäftigte, Arbeitnehmer, Gewerkschaften, Mitarbeiter, Betriebsräte, salarié-e-s, employé-e-s, werknemers, medewerkers,ondernemingsraden	
Workers	Original category	Handwerker, Geringqualifizierte, LKW-Fahrer, Prostituierte, vrachtwagenchauffeur	
Socio-cultural prof.	Based on string variable identifying terms such as "teacher" or "doctors"	Lehrer, Forscher/Wissenschaftler, Pflegekräfte, Ärzte, leraren/leerkrachten, wetenschappers, zorgverleners, artsen	
Poor People	Collapsing the original categories of "poor people" and "welfare recipients"	Arbeitslose, Erwerbslose, Geringverdiener, einkommensschwache Haushalte, lage inkomens,	
Rich People	Original category		Reiche, Wohlhabenden, hoge inkomens
Students	Based on string variable to identify them in the broader original category of "academia" with terms such as "students"		
Women	Original category	Frauen, femmes, vrouwen	
LGBT people	Collapsing of original categories of "homosexual people", "transgender people"	Homosexuelle, gleichgeschlechtliche Paare, Transgender, Intersexuelle, homoseksuelen, transgenders	

Immigrants	Collapsing of categories of „asylum seekers", "people with migrant background" and "foreign nationals"	Migrantinnen und Migranten, Menschen mit Migrationshintergrund, Einwanderer, Flüchtlinge, Asylsuchende, migranten, vluchtelingen, asielzoekers,	(gewaltätige) Ausländer, étrangers (criminels), (faux) réfugiés, allochtonen, migranten
